# **A meta-analysis uncovers the first sequence variant conferring risk of Bell**’**s palsy**

**DOI:** 10.1038/s41598-021-82736-w

**Published:** 2021-02-18

**Authors:** Astros Th. Skuladottir, Gyda Bjornsdottir, Gudmar Thorleifsson, G. Bragi Walters, Muhammad Sulaman Nawaz, Kristjan Helgi Swerford Moore, Pall I. Olason, Thorgeir E. Thorgeirsson, Brynja Sigurpalsdottir, Gardar Sveinbjornsson, Hannes P. Eggertsson, Sigurdur H. Magnusson, Asmundur Oddsson, Anna Bjornsdottir, Arnor Vikingsson, Olafur A. Sveinsson, Maria G. Hrafnsdottir, Gudrun R. Sigurdardottir, Bjarni V. Halldorsson, Thomas Folkmann Hansen, Helene Paarup, Christian Erikstrup, Kaspar Nielsen, Mads Klokker, Mie Topholm Bruun, Erik Sorensen, Karina Banasik, Kristoffer S. Burgdorf, Ole Birger Pedersen, Henrik Ullum, Ingileif Jonsdottir, Hreinn Stefansson, Kari Stefansson

**Affiliations:** 1 deCODE Genetics/Amgen Inc, Reykjavik, Iceland; 2 Faculty of Medicine, University of Iceland, Reykjavik, Iceland; 3 University of Reykjavik, Reykjavik, Iceland; 4 Laeknasetrid Clinic, Reykjavik, Iceland; 5 Landspitali - the National University Hospital of Iceland, Reykjavik, Iceland; 6 Kobenhavns Universitet, Kobenhavn, Denmark; 7 Odense Universitetshospital, Odense, Denmark; 8 Aarhus Universitetshospital, Aarhus, Denmark; 9 Aalborg Universitetshospital, Aalborg, Denmark; 10 Rigshospitalet, Kobenhavn, Denmark; 11 Neastved Hospital, Neastved, Denmark

**Keywords:** Genetics, Molecular biology, Neuroscience, Diseases, Neurology, Pathogenesis

## Abstract

Bell’s palsy is the most common cause of unilateral facial paralysis and is defined as an idiopathic and acute inability to control movements of the facial muscles on the affected side. While the pathogenesis remains unknown, previous studies have implicated post-viral inflammation and resulting compression of the facial nerve. Reported heritability estimates of 4–14% suggest a genetic component in the etiology and an autosomal dominant inheritance has been proposed. Here, we report findings from a meta-analysis of genome-wide association studies uncovering the first unequivocal association with Bell’s palsy (rs9357446-A; *P* = 6.79 × 10^−23^, OR = 1.23; *N*_cases_ = 4714, *N*_controls_ = 1,011,520). The variant also confers risk of intervertebral disc disorders (*P* = 2.99 × 10^−11^, OR = 1.04) suggesting a common pathogenesis in part or a true pleiotropy.

## Introduction

Bell’s palsy, also known as idiopathic facial paralysis, is an acute or subacute weakness of the facial muscles innervated by the seventh cranial nerve (the facial nerve). Symptoms are usually unilateral although both sides can be affected. Although symptoms usually subside within one month, up to 30% of cases show delayed or incomplete recovery with mild to severe residual facial weakness, contracture, and/or hemifacial spasm^1^. Other symptoms vary from person to person and may include mild facial pain, numbness or stiffness and headache. The annual incidence is about 20–30 cases per 100,000, with about 1 in 60 lifetime risk. The disorder usually occurs in individuals aged 15–40 and affects men and women equally^2^. Risk factors include hypertension^3^, diabetes^4^, immunosuppression^5^, Lyme disease^6^, and in rare cases inflammatory demyelinating neuropathy such as Guillain-Barré syndrome^7^.

While the exact etiology of Bell’s palsy is unknown, it has been suggested that the disorder is caused by inflammation or compression of the seventh cranial nerve^6^. The literature has highlighted several viral illnesses such as herpes simplex type 1, which has been detected in 31–79% of cases^8,9^, human immunodeficiency viruses^10^, varicella-zoster, and Epstein-Barr^11^. After a primary infection, these latent viruses reside in the geniculate ganglion or in lymphocytes. At times with e.g. stress, the viruses may be reactivated and cause local damage to the myelin^8^. The two most common treatments are corticosteroids and antivirals. However, studies have found that antivirals alone are not helpful and antiviral treatment in combination with corticosteroids have conflicting results^12^. Although the heritability has been estimated to be only 4–14%^1,13^, numerous familial cases have been reported.

Here, we report the first meta-analysis of genome-wide association studies (GWASs) of Bell’s palsy, combining data from Iceland (deCODE genetics), the UK (UK Biobank), Denmark (Danish Blood Donor Study [DBDS] and Copenhagen Hospital Biobank [CHB]), and Finland (FinnGen), which uncovers an association with a common intergenic variant.

## Results

### Genome-wide meta-analysis

**Figure 1:**
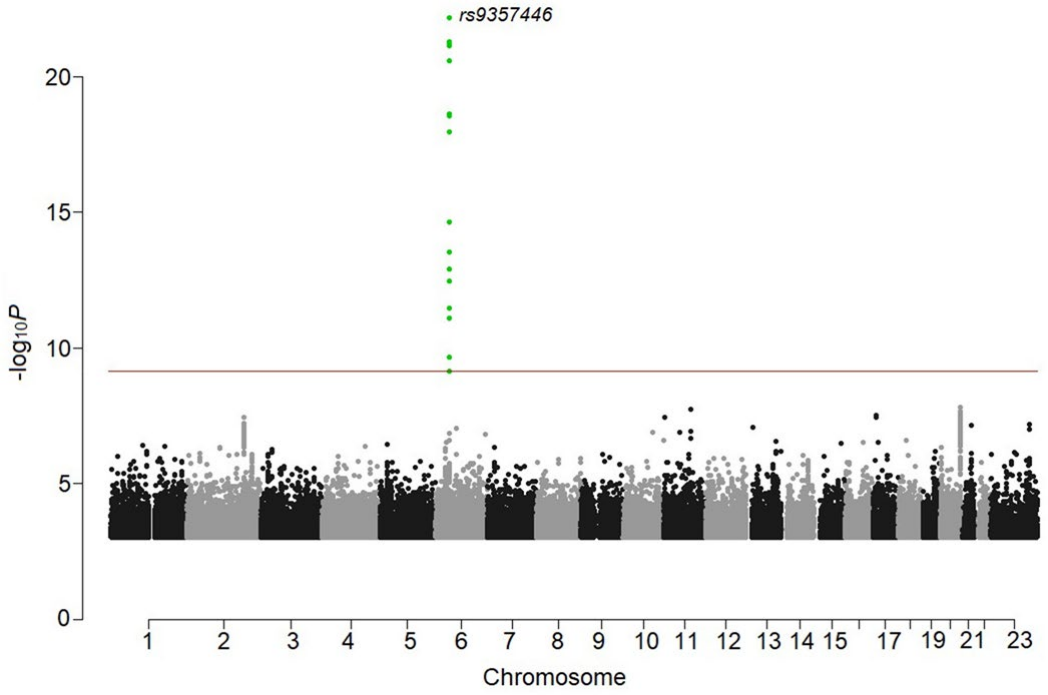
Manhattan plot for the Bell’s palsy meta-analysis. One genome-wide significant locus was detected. The − log_10_ *P-*values (y-axis) are plotted for each variant against their chromosomal position (x-axis). The red line denotes the significance threshold for intergenic variants, *P* ≤ 7.4 × 10^–10^. *P*-values are two sided and derived from a likelihood ratio test (Methods). Individual Manhattan plots for each dataset are shown in Supplementary Fig. 2.

We performed a meta-analysis combining Bell’s palsy GWAS results for 30,341,632 variants from Iceland (*N*_cases_ = 290, *N*_controls_ = 342,122), the UK (*N*_cases_ = 2024, *N*_controls_ = 406,541), Denmark (*N*_cases_ = 1383, *N*_controls_ = 141,497), and Finland (*N*_cases_ = 1017, *N*_controls_ = 121,360). Bell’s palsy cases were assigned ICD-10 (G51.0) diagnoses by a physician in all study cohorts (Methods; demographics in Supplementary Table 1). Applying logistic regression, we identified an association with a common variant, rs9357446-A (Allele Frequency [AF]_ICE_ = 55.5%, AF_UK_ = 53.3%, AF_DEN_ = 52.7%, AF_FIN_ = 50.8%), that confers risk of Bell’s palsy (*P* = 6.79 × 10^–23^, Odds ratio [OR] = 1.23) (Table 1, Fig. 1, Supplementary Data). The full genotypic model does not significantly differ from the additive model (*P*_full vs. additive_ = 0.19; Supplementary Fig. 1).

**Table 1:** Association of a novel, genome-wide significant variant identified in the Bell’s palsy meta-analysis.

Position (Hg38)	rs-name	Allele (EA/OA)	EAF (%)	Gene/[Locus]	Coding effect	OR (95% CI)	*P*	*P-het*	*P* random effect
chr6:44,479,861	rs9357446	A/G	53.1	[CDC5L]	Intergenic	1.23 (1.18, 1.28)	6.79 × 10^–23^	0.048	6.83 × 10^–23^

Significance levels and effects are shown for the combined analysis. *EA* effect allele, *OA* other allele, *EAF* effect allele frequency, OR odds ratio, *CI* confidence interval, *P-het P*-value for test of heterogeneity between Iceland, the UK, Denmark, and Finland.

There is a small evidence of heterogeneity (*P*-het ≤ 0.05) describing a modest difference in the four datasets. Therefore, we applied a random-effects model which assumes that there may be different underlying true effects estimated in each dataset. The variant maintains a weighted Bonferroni significance (*P* = 6.83 × 10^–23^) based on predicted functional impact of the association signal.

**Figure 2:**
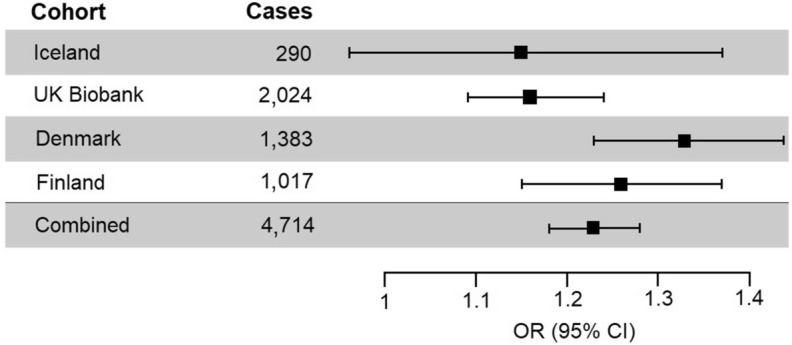
Forest plot of the observed risk of Bell’s palsy for individuals carrying rs9357446-A in each study sample. The number of Bell’s palsy cases in each sample is shown in the second column.

The risk of developing Bell’s palsy is similar in all datasets (See *P-het* in Table 1, Fig. 2). The estimated genetic inflation is low for the datasets (Iceland = 1.03, UK = 1.01, Denmark = 1.00, Finland = 1.03) considering that both the Icelandic and the UK samples have a high number of related individuals. In Iceland, a large fraction of the population has participated in genetic studies and in the UK Biobank, ~ 30% of the participants have a relative of third degree or closer^14^.

**Figure 3:**
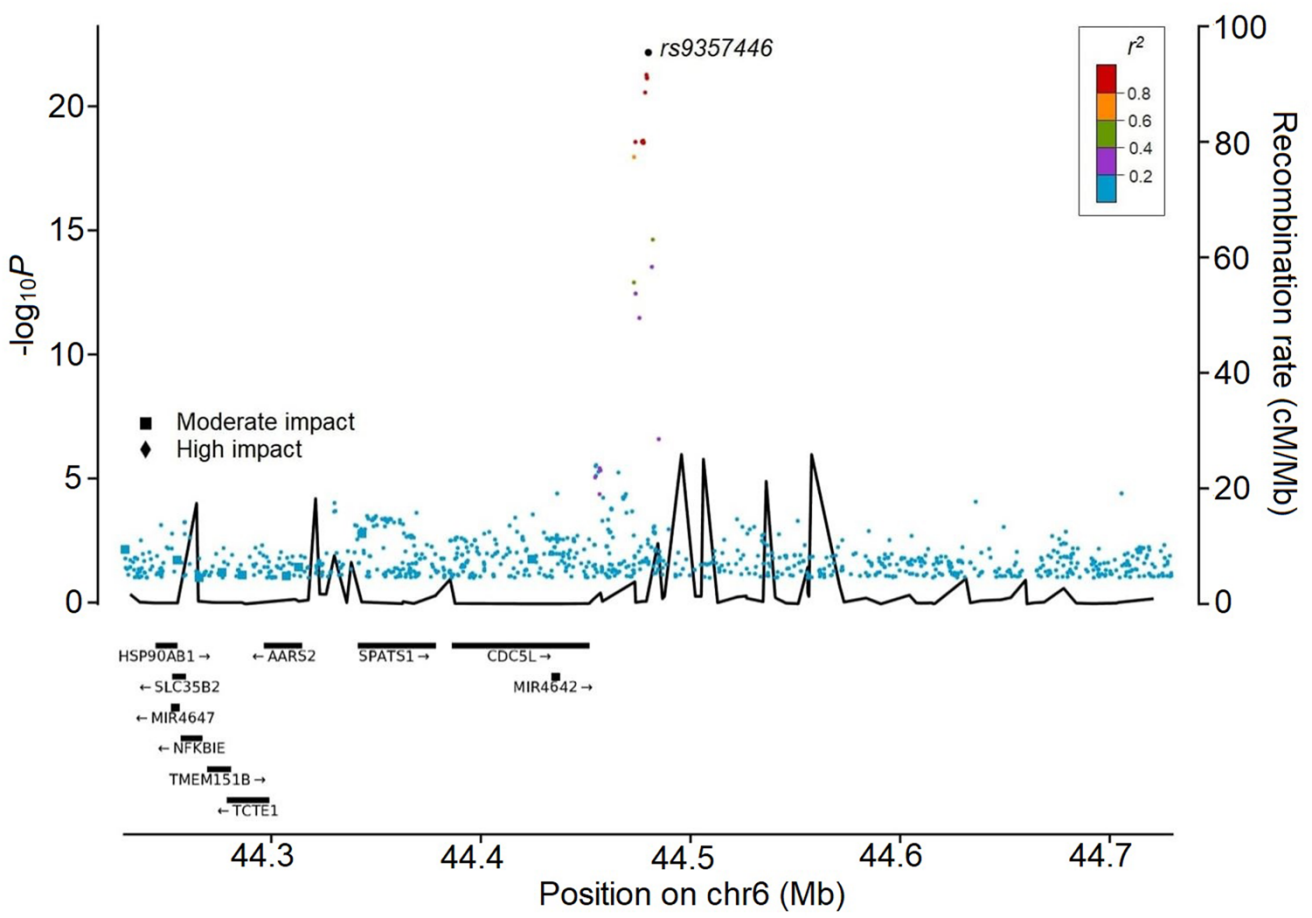
Regional visualization of the Bell’s palsy meta-analysis. The leading variant is colored in black. Other variants are colored by the degree of correlation (*r*^2^) with rs9357446. The − log_10_ *P*-values on the left y-axis (two-sided logistic regression) are plotted for each variant against their chromosomal position (x-axis). The right y-axis shows calculated recombination rates based on the Icelandic data at the chromosomal location, plotted as solid black lines.

Based on the Icelandic data, twenty-two non-coding variants in the region correlate with rs9357446 (*r*^2^ > 0.2) and of those, eleven are highly correlated (*r*^2^ > 0.9; Fig. 3, Supplementary Table 2). A conditional analysis demonstrates that the association of these variants with Bell’s palsy is accounted for by rs9357446, indicating one independent variant. The variant is not correlated (*r*^2^ < 0.2) with any known coding or structural variants.

We examined *cis*-eQTL in blood and adipose tissue in Iceland and did not find an association between rs9357446 and expression of nearby genes (Supplementary Note). The variant did not affect expression in any nearby genes in 18 other databases listed in Supplementary Table 3. We tested associations between rs9357446 and 4792 plasma proteins measured in 35,559 Icelanders but did not find a significant association (Supplementary Note). We also measured methylation of CpGs in a 2000 Bp window around rs9357446. A CpG methylation at chr6:44,480,140 is slightly increased in rs9357446-A carriers (*N* = 1236, *P* = 0.024). However, this CpG is generally methylated and a slight increase in methylation is not likely to have any biological effect.

We performed genetic correlation analyses using the Bell’s palsy meta-analysis and 600 GWASs from the UK Biobank^15^. The Bell’s palsy meta-analysis associates with seven lifestyle traits (*P* ≤ 0.05/600 = 8.33 × 10^–5^; Supplementary Table 4) such as number of treatments or medications taken (rG = 0.41, *P* = 1.55 × 10^–8^) and poor health (rG =  − 0.36, *P* = 1.79 × 10^–7^).

### rs9357446-A and associations with other phenotypes

The variant has previously been reported to associate with decreased lung function measured by forced vital capacity (FVC)^16^. This finding replicates in the Icelandic dataset (*P* = 0.0145, *β* =  − 0.037). We did not find association with other lung related phenotypes including asthma, chronic obstructive pulmonary disease, or tuberculosis combining the Icelandic, UK and Danish data in meta-analyses and we did not find associations with other reported risk factors such as hypertension and type 1 diabetes (Supplementary Table 5). In a meta-analysis of 58,854 cases and 922,958 controls from Iceland, the UK, Denmark, and Finland, rs9357446-A associates with intervertebral disc disorders (IDD [ICD-10: M51]; *P* = 2.99 × 10^–11^, OR = 1.04; unpublished work by Bjornsdottir G et al*.*). rs9357446-A is among the top variants at the locus for IDD (Supplementary Fig. 3).

## Discussion

The precise pathophysiology of Bell’s palsy is unknown and biologically targeted treatment is lacking. The aim of the study was to search for variations in the human genome affecting risk of Bell’s palsy and thus use genetics to uncover biological underpinnings of this somewhat mysterious disease. Here, we report the first unequivocal association between Bell’s palsy and a sequence variant, rs9357446-A (*P* = 6.79 × 10^–23^, OR = 1.23), in a meta-analysis of four study cohorts.

While rs9357446-A confers risk of Bell’s palsy, it also confers low risk of IDD as well as reduced lung function measured by decreased FVC^16^ suggesting shared etiology of these diverse diseases that are all but certain to differ in the pathogenesis. The common variant at 6p21.1 is intergenic. Nearby genes are *LOC105375075*, *CDC5L*, *MIR4642*, *SPATS1*, *AARS2*, *TCTE1*, *TMEM151B,* and *SLC35B2*. Our transcript- and proteomics analyses did not pinpoint a Bell’s palsy gene at this locus. Although uncorrelated with rs9357446 (*r*^2^ < 0.2), several sequence variants in the closest gene, cell division cycle 5 like (*CDC5L*), have been found to associate with related phenotypes, including osteoarthritis^17^, bone density^18^, ossification of the spine^19^, and lung function measured by FVC and forced expiratory volume^16^. Another potential candidate at the locus, *SLC35B2,* is a key component of a protein sulfation pathway where it plays a part in modifying C–C Motif Chemokine Receptor 5 (CCR5), that is one of HIV’s host proteins for entry into the cell^20^. Several other studies point to the importance of CCR5 in determining disease severity of other viral infections in animal models^21,22^. In addition, *SLC35B2* is involved in proteoglycan synthesis. Cartilage is particularly rich in proteoglycans, and changes in the structure and composition of glycosaminoglycans, forming the sugar chain attached to the protein core of proteoglycans, have been found in osteoarthritis. Mutations in *slc35b2* in zebrafish have been shown to cause deformation of the craniofacial skeleton^23^.

Bell’s palsy is still idiopathic since we do not know what causes it; there are no biomarkers to support the diagnosis and the diagnosis requires exclusion of any other cause of facial paralysis^24^. In Bell’s palsy, a viral infection is considered one of the possible causes^8^. The main treatment is corticosteroids, which supports the theory of edema induced entrapment neuropathy. The facial nerve traverses through the temporal bone (the facial canal) and branches to innervate the facial muscles. The narrowest part of the canal, the labyrinthine segment, is where most cases of compression occur, which may result in ischemic neuropathy of the facial nerve^25^.

We were unable to determine a gene at 6p21.1 involved in Bell’s palsy pathology. However, the association of rs9357446-A with IDD brings up the possibility that the variant may confer risk of Bell’s palsy through an effect on cartilage and bone development. Alternatively, the association between Bell’s palsy, IDD, and reduced lung function may be a result of a true pleiotropy. Although more studies are needed, the identification of this variant contributes to the limited understanding of Bell’s palsy.

## Methods

### Ethics statement

All Icelandic data were collected through studies approved by the National Bioethics Committee (NBC; License #VSN-12-162 with amendments) following review by the Icelandic Data Protection Authority. Participants donated blood or buccal samples after signing a broad informed consent allowing the use of their samples and data in all projects at deCODE genetics approved by the NBC. All personal identifiers of the participants' data were encrypted by a third-party system, approved and monitored by the Icelandic Data Protection Authority.

The UK Biobank data were obtained under application number 24898. All phenotype and genotype data were collected following an informed consent obtained from all participants. The North West Research Ethics Committee reviewed and approved UK Biobank’s scientific protocol and operational procedures (REC Reference Number: 06/MRE08/65).

The Danish Data Protection Agency (2007-58-0015) and the National Committee on Health Research Ethics (NVK-1700407) approved the genetic study on Danish Blood Donor Study (DBDS). The data requested for this study was approved by the DBDS steering committee. Samples from the Copenhagen Hospital Biobank (CHB) were included as part of the study on pain related diseases under the genetics of pain and degenerative musculoskeletal disease protocol (NVK-1803012).

Samples and phenotype data were collected from the Finnish biobanks and the national health registers, respectively, for the FinnGen database. The Coordinating Ethics Committee of the Helsinki and Uusimaa Hospital District evaluated and approved the FinnGen research project. The project complies with existing legislation (in particular the Biobank Law and the Personal Data Act). The official data controller of the study is University of Helsinki.

### Study subjects

To identify Bell’s palsy cases in Iceland, we searched for patients over the age of 18 with International Classification of Diseases (ICD-10) diagnosis code G51.0 and ICD-9 code 351.0 at the National University Hospital of Iceland and all Icelandic primary and secondary care centers. Records spanned from 1983 to 2019.

The UK Biobank study is a large prospective cohort study of ~ 500,000 individuals in the age range of 40–69 from across the UK. Extensive phenotype and genotype data have been collected for participants, including ICD diagnosis codes. Bell’s palsy diagnoses in the UK Biobank were obtained by searching for cases with ICD-10 code G51.0 from UK Biobank General Practice clinical event records (Field ID 42,040) and hospital diagnoses (Field ID 41,270 and 41,271). Only individuals of Caucasian ancestry were included.

The CHB is a research bank, which contains left over samples from diagnostic procedures on in- and outpatients in hospitals in the Danish Capital Region. The DBDS GWAS study is a nationwide prospective cohort study of ~ 100,000 blood donors. We applied for ICD-10 code G51.0 from CHB and DBDS^26^. Genetic ancestry quality control was performed in two stages. Firstly, ADMIXTURE v1.23^27^ was run in supervised mode with 1000G populations CEU, CHB, and YRI^28^ as training samples and Danish individuals as test samples. Input data for ADMIXTURE had long-range LD regions removed^29^ and was then LD-pruned with PLINK v.190b3a^30^ –indep-pairwise 200 25 0.3. Danish samples with less than 0.9 assigned CEU ancestry were excluded. In the second stage, remaining Danish samples were projected onto a principal component analysis (PCA), calculated with in-house tools on an in-house European reference panel. The UMAP package in R^31^ was used to reduce the coordinates of test samples on 20 principal components to two dimensions. Additional European samples not in the original reference, were also projected onto the PCA and UMAP to help identify the ancestries represented in clusters. A polygon informed by visual inspection was drawn to include all samples with very similar ancestries to the main Danish cluster.

The phenotype data from the FinnGen study was produced from several national health registries. All Bell’s palsy cases were diagnosed by a physician and categorized using ICD-10 code G51.0, ICD-9 code 351.0, and ICD-8 code 350. The summary statistics for available phenotypes, including Bell’s palsy, were imported on June 16th 2020 from a source available to consortium partners (version 3; http://r3.finngen.fi).

We combined 290 cases and 342,122 controls from Iceland, 2024 cases and 406,541 controls from the UK, 1383 cases compared to 41,497 patients with other pain related disorders (CHB) and 100,000 controls (DBDS) from Denmark, and 1017 cases and 121,360 controls from Finland; in total, 4714 cases and 1,011,520 controls.

### Genotyping

The preparation of samples and the whole-genome sequencing of 49,962 Icelanders has been described in detail elsewhere^32,33^. In short, 37.6 million high-quality sequence variants were identified by sequencing 49,962 Icelanders using GAIIx, HiSeq, HiSeqX, and NovaSeq Illumina technology to a mean depth of at least 17.8 × . SNPs and indels were identified and their genotypes called using joint calling with Graphtyper^34^. Additionally, over 165,000 Icelanders (including all sequenced Icelanders) have been genotyped using various Illumina SNP chips and phased using long-range phasing^35^, which allows for improving genotype calls using the information about haplotype sharing. The genotypes of the high-quality sequence variants were imputed into the chip-typed Icelanders^36^. To increase the sample size and power to detect associations, the sequence variants were also imputed into relatives of the chip-typed using genealogic information. All the tested variants had imputation information over 0.8.

The UK Biobank samples were genotyped with a custom-made Affymetrix chip, UK BiLEVE Axiom in the first 50,000 individuals^37^, and the Affymetrix UK Biobank Axiom array^38^ in the remaining participants. Imputation was performed by the Wellcome Trust Centre for Human Genetics using a combination of the Haplotype Reference Consortium^39^, UK10K haplotype resources^40^, and 1000Genomes phase 3 panels^28^. A total of 96 million variants have been imputed.

Over 332,000 samples from the CHB and DBDS together with ~ 238,000 genotyped samples from Northwestern Europe were long-range phased using Eagle2^41^. Samples and variants with less than 98% yield were excluded. We used the same methods as used for the Icelandic data^32,35^ to create a haplotype reference panel by phasing the whole-genome sequence genotypes (*N* = 8635) using the phased chip data (*N* = 332,949), and to impute the genotypes from the haplotype reference panel into the phased chip data. Whole genome sequencing, chip-typing, quality control, and the subsequent imputation from which the data for this analysis were generated was performed at deCODE genetics.

A custom-made FinnGen ThermoFisher Axiom array (> 650,000 SNPs) was used to genotype ~ 135,600 FinnGen samples at Thermo Fisher genotyping service facility in San Diego. Imputation was performed using the Finnish population specific WGS backbone. More than 16 million variants have been imputed.

### Association analysis

We performed logistic regression using the Icelandic, UK, and Danish data and combined the results with imported association results from Finland to test for association between sequence variants and Bell’s palsy using software developed at deCODE genetics^32^. We used LD score regression to account for distribution inflation due to cryptic relatedness and population stratification in the Icelandic, UK, and Danish data^42^. In the Icelandic association analysis, we adjusted for sex, county of origin, current age or age at death (first and second order term included), blood sample availability for the individual, and an indicator function for the overlap of the lifetime of the individual with the time span of phenotype collection. In the UK association analysis, we adjusted for sex, age, and the first 40 principal components to adjust for population stratification. In the Danish association analysis, we adjusted for sex, whether the individual had been chip-typed and/or sequenced, and the first 20 principal components. The FinnGen association analysis was adjusted for sex, age, the genotyping batch, and the first 10 principal components.

For the meta-analysis, we used a fixed-effects inverse variance method^43^ to combine the results from the four study groups in which the groups were assumed to have a common OR but allowed to have different population frequencies for alleles and genotypes. Variants with imputation information below 0.8 were excluded from the analysis. Sequence variants were mapped to NCBI Build38 and matched on position and alleles to harmonize the four datasets. We estimated the genome-wide significance threshold and corrected for multiple testing with a Bonferroni procedure weighted for variant classes and predicted functional impact^44^. A likelihood ratio test was performed for the genome-wide significant variant to test the heterogeneity of the effect estimate in the four datasets. The null hypothesis is that the effects are the same in all datasets and the alternative hypothesis is that the effects differ between datasets.

Conditional analysis was performed to identify the most likely causal variant at the locus and to identify any secondary signals. We used the true genotypes of participants in the study from Iceland, the UK, and Denmark. LD between variants was estimated using a set of 8700 WGS Icelanders and was restricted to variants within one Mb from the index variant.

Genetic correlation analyses between the Bell’s palsy meta-analysis and 600 published GWAS traits (*P* ≤ 0.05/600 = 8.33 × 10^–5^) from the UK Biobank^15^ with effective sample size over 5000 were performed using LDSC method^42,45^. The LDSC method suggests minimal effective sample size of 5000 for a GWAS trait to get unbiased estimates of genetic correlation and heritability. Since the published GWAS studies used in the analysis have Caucasian ancestry, we used pre-computed LD scores from a 1000 genome panel with r^2^ from HapMap3 excluding HLA region. The HLA region was excluded for its complex genetic pattern and associations with wide number of traits. Moreover, the default parameters of LDSC method were used to compute genetic correlation and heritability estimates.

### Transcriptomics

RNA sequencing of 14,248 genes was performed on the whole blood of 13,175 Icelanders and of 9396 genes on the subcutaneous adipose tissue from 700 Icelanders. Gene expression was computed based on personalized transcript abundances^46^. Association between variants and gene expression was estimated using a generalized linear regression, assuming additive genetic effect and quantile normalized gene expression estimates, adjusting for measurements of sequencing artefacts, demographic variables, blood composition, and hidden covariates^47^.

### Proteomics

We used SomaLogic SOMAscan proteomics assay to test the association of the sequence variant with protein levels in plasma. The assay scanned 4983 proteins in samples from 35,559 Icelanders with genetic information available at deCODE genetics. Plasma protein levels were standardized and adjusted for year of birth, sex, and year of sample collection (2000–2019).

### Methylomics

We performed whole-methylome-sequencing on 4133 samples using ONT Ligation Sequencing Kit (SQK-LSK108) or Rapid Sequencing Kit (SQK-RAD003)^48^. MinION and PromethION devices with FLO-106/R9.4 flow cells were used for base calling. Methylation on CpGs was called using Nanopolish (version 0.6.1)^49^.

## Supplementary information

Supplementary data.

Supplementary information.

## Data Availability

The sequence variants from the Icelandic population whole-genome sequence data have been deposited at the European Variant Archive under accession code PRJEB15197. The authors declare that the data supporting the findings of this study are available within the article, its Supplementary Information file, and upon reasonable request.
