# Supplementary material for: A meta-analysis uncovers the first sequence variant conferring risk of Bell’s palsy
Source: Sci Rep. 2021 Feb 18;11:4188. doi: 10.1038/s41598-021-82736-w (PMC7893061; doi:10.1038/s41598-021-82736-w)
Supplement: Supplementary file 2 — Supplementary information. [file 41598_2021_82736_MOESM2_ESM.pdf]

Supplementary information

A meta-analysis uncovers the first sequence variant conferring risk of Bell's palsy

Astros Th. Skuladottir, Gyda Bjornsdottir, Gudmar Thorleifsson, G. Bragi Walters, Muhammad Sulaman Nawaz, Kristjan Helgi Swerford Moore, Pall I. Olason, Thorgeir E. Thorgeirsson, Brynja Sigurpalsdottir, Gardar Sveinbjornsson, Hannes P. Eggertsson, Sigurdur H. Magnusson, Asmundur Oddsson, Anna Bjornsdottir, Arnor Vikingsson, Olafur A. Sveinsson, Maria G. Hrafnsdottir, Gudrun R. Sigurdardottir, Bjarni V. Halldorsson, Thomas Hansen, Helene Paarup, Christian Erikstrup, Kaspar Nielsen, Mads Klokker, Mie Topholm Bruun, Erik Sorensen, Karina Banasik, Kristoffer S. Burgdorf, Ole Birger Pedersen, Henrik Ullum, Ingileif Jonsdottir, Hreinn Stefansson, and Kari Stefansson

Contents

Supplementary Figure 1. Genotypic effect of rs9357446 2

Supplementary Figure 2. Manhattan plot for each dataset 3

Supplementary Figure 3. Locus plot for intervertebral disc disorders 4

Supplementary Table 1. Demographics for each GWAS sample in the meta-analysis 5

Supplementary Table 2. Correlated variants with rs9357446 6

Supplementary Table 3. eQTL databases 7

Supplementary Table 4. Association results from genetic correlation analysis 8

Supplementary Table 5. Association results of rs9357446 with other diseases 9

Supplementary Note. rs9357446-A and RNA expression 10

Supplementary Note. rs9357446-A and plasma proteomics 11

Supplementary Figure 1. Genotypic effect of rs9357446

The odds ratio (y-axis) difference between carrier status (x-axis); non-carriers (rs9357446-GG), heterozygotes (rs9357446-AG), and homozygotes (rs9357446-AA). The whiskers represent 95% confidence interval.

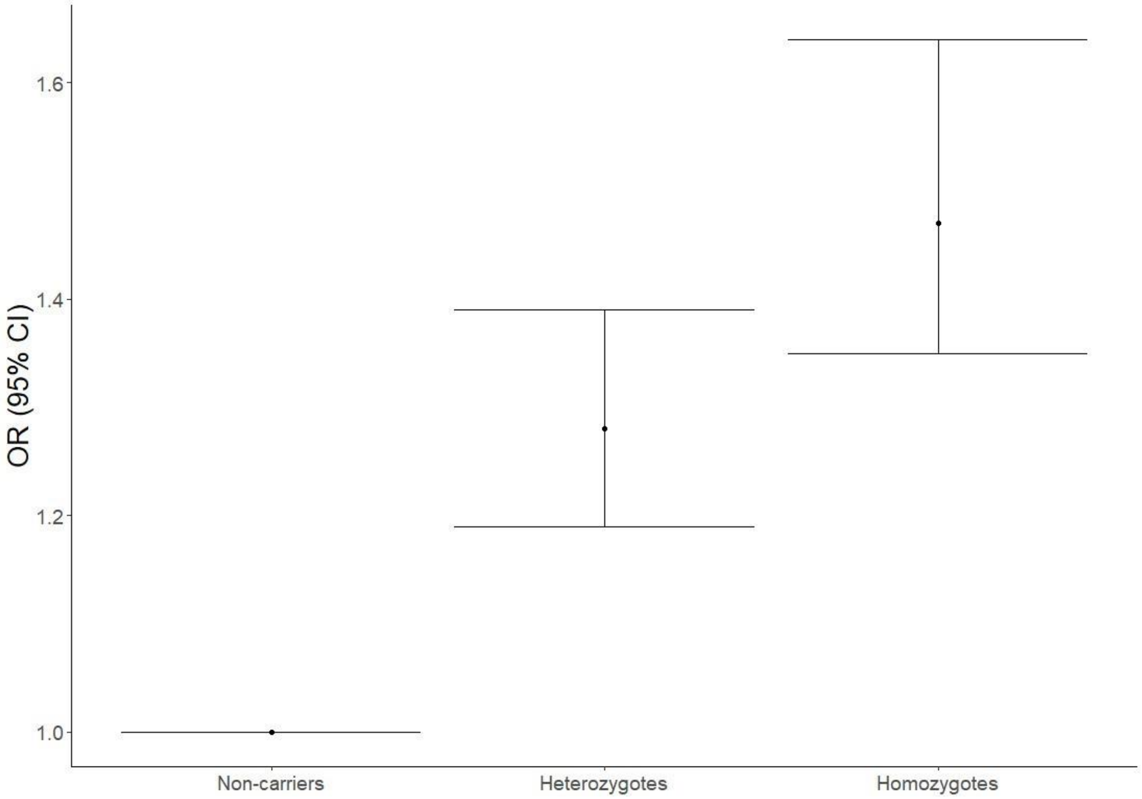

Supplementary Figure 2. Manhattan plot for each dataset

Individual manhattan plots for each dataset. a) Iceland (deCODE genetics), b) the UK (UK Biobank), c) Denmark (Danish Blood Donor Study and Copenhagen Hospital Biobank), and d) Finland (Finngen). The -$\log_{10}P$-values (y-axis) are plotted for each variant against their chromosomal position (x-axis). The red line denotes the significance level of intergenic variants, $P \leq 7.4 \times 10^{-10}$. P values are two sided and derived from a likelihood ratio test (Methods). The variants colored green are the significant variants in the combined meta-analysis.

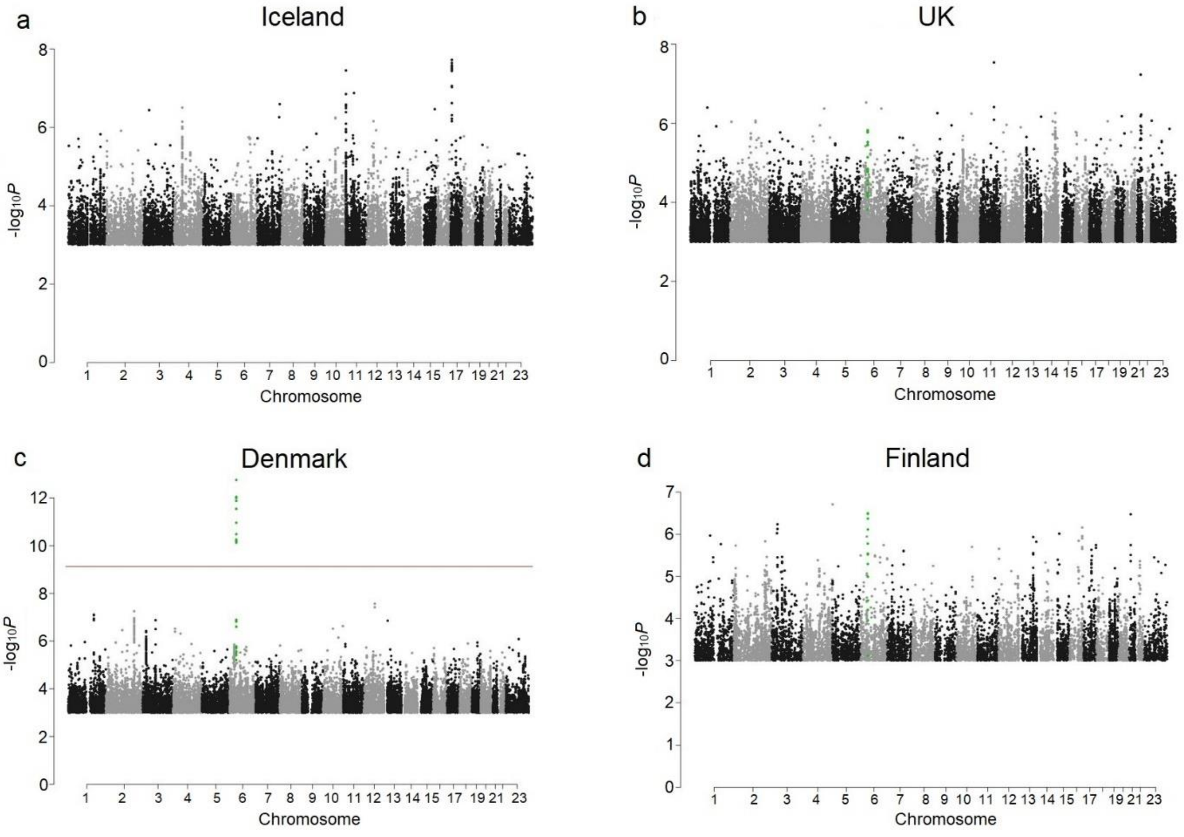

Supplementary Figure 3. Locus plot for intervertebral disc disorders

Locus plot using meta-analysis data from Iceland and the UK at the *CDC5L* locus. Variants are colored by the degree of correlation (r^2) with rs9357446, which is shown as a black dot. The -log$_{10}$*P*-values on the left y-axis (two-sided logistic regression) are plotted for each variant against their chromosomal position (x-axis). The right y-axis shows calculated recombination rates at the chromosomal location, plotted as solid black lines.

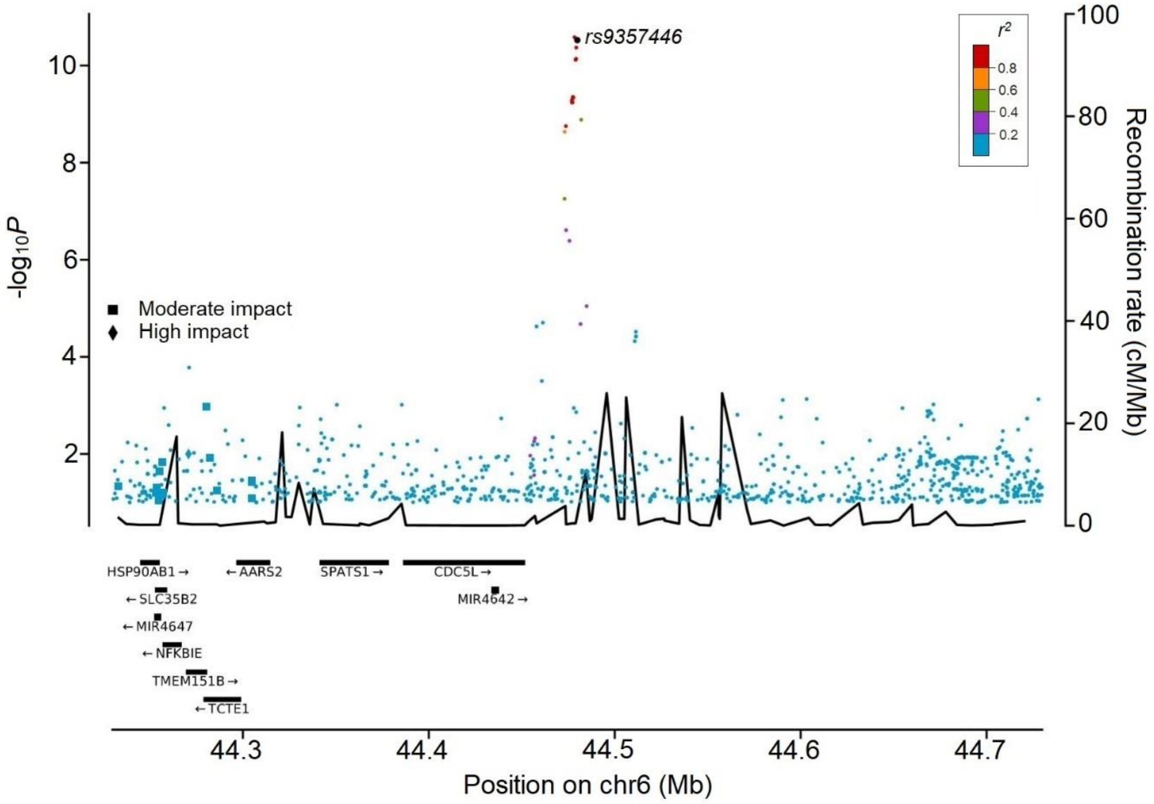

Supplementary Table 1. Demographics for each GWAS sample in the meta-analysis.

M mean, *SD* standard deviation.

		***N* (%)**	**Age at first event, M (SD)**
**ICE**	Males	117 (40.3)	46.2 (24.5)
	Females	173 (59.6)	47.3 (24.2)
**UK**	Males	983 (48.6)	55.4 (14.5)
	Females	1,041 (51.4)	53.8 (15.4)
**DNK**	Males	750 (54.2)	52.6 (17.0)
	Females	633 (45.8)	51.9 (19.9)
**FIN**	Males	440 (43.3)	52.9
	Females	557 (54.8)	50.6

Supplementary Table 2. Correlated variants with rs9357446

Twenty-two variants are correlated with rs9357446 (r^2 > 0.2). Eleven variants are highly correlated (r^2 > 0.9). The $-\log_{10}P$-values show association with Bell's palsy in the meta-analysis of Icelandic, the UK, Danish and Finnish data.

rsName	Position	Effect allele	Other allele	EAF (%)	r^2	D'	-log10(*P*)
rs9357446	chr6:44479861	G	A	44.2	1.00	1.00	22.2
rs7770012	chr6:44479182	C	A	42.4	0.93	1.00	21.1
rs7770034	chr6:44479267	A	G	42.2	0.92	1.00	21.1
rs2281690	chr6:44478937	G	A	42.2	0.92	1.00	21.3
rs6929734	chr6:44478351	G	T	41.5	0.88	0.99	20.6
rs4714791	chr6:44473703	T	G	44.2	0.83	0.91	18.6
rs9296437	chr6:44477359	A	G	43.1	0.82	0.92	18.6
rs9395026	chr6:44476894	T	A	43.1	0.82	0.92	18.6
rs9296435	chr6:44476966	T	C	43.1	0.82	0.92	18.6
rs9296438	chr6:44477438	A	G	43.1	0.82	0.92	18.5
rs9367197	chr6:44477709	T	C	43.1	0.82	0.92	18.5
rs9296436	chr6:44477309	A	G	43.1	0.82	0.92	18.5
rs9349288	chr6:44473044	C	T	46.2	0.75	0.90	18.0
rs12154055	chr6:44481960	G	A	65.3	0.48	0.84	14.6
rs9395021	chr6:44472985	C	T	57.0	0.44	0.86	12.9
rs911983	chr6:44473815	T	C	67.5	0.32	0.92	12.4
rs10948139	chr6:44481583	C	T	60.6	0.30	0.77	13.5
rs12664617	chr6:44484869	C	T	74.8	0.30	0.84	6.59
rs9395025	chr6:44475625	G	A	68.0	0.24	0.80	11.5
rs566078	chr6:44454514	G	A	64.7	0.22	0.71	5.04
rs543844	chr6:44457063	A	G	65.8	0.20	0.70	5.33
rs517214	chr6:44456639	A	T	65.9	0.20	0.70	5.42
rs11376630	chr6:44456591	A	AT	65.9	0.20	0.70	4.37

Supplementary Table 3. eQTL databases

Source	#/Type of Tissues	#Individuals	#Genes/Type of variant	Website
GTEx v8	49	650	16.729	https://gtexportal.org/home/
Vosa et al.	Blood	31.000	13.195	https://www.biorxiv.org/content/10.1101/447367v1
Strunz et al. (2018)	Liver	588	1.299	https://www.nature.com/articles/s41598-018-24219-z
Ratnapriya et al. (2019)	Eye	453	5.160	https://www.nature.com/articles/s41588-019-0351-9
Ng et al. (2017)	Brain	494	3.041	https://www.nature.com/articles/nn.4632
Hauberg et al. (2017)	8	550	1.078	https://www.cell.com/ajhg/fulltext/S0002-9297(17)30161-1
Zeller et al. (2010)	Momocytes	1.490	2.745	https://pubmed.ncbi.nlm.nih.gov/20502693/
Liang et al. (2013)	Lymphoblastoid	950	-	https://pubmed.ncbi.nlm.nih.gov/23345460/
Hao et al. (2012)	Lung	1.111	4.932	https://journals.plos.org/plosgenetics/article?id=10.1371/journal.pgen.1003029
Gillies et al. (2018)	Kidney (glomerulus)	187	557	https://www.ncbi.nlm.nih.gov/pmc/articles/PMC6081280/
Gillies et al. (2018)	Kidney (tubulointerstitial)	187	187	https://www.ncbi.nlm.nih.gov/pmc/articles/PMC6081280/
Pala et al. (2017)	White blood cells	624	7.679	https://www.nature.com/articles/ng.3840
Yao et al. (2017)	Whole blood	5.257	2.457	https://www.sciencedirect.com/science/article/pii/S0002929717300708
Franzen et al. (2016)	7	600	1.823	https://science.sciencemag.org/content/353/6301/827.long
Lee et al. (2014)	Dentritic cells (rested and stimulated)	534	171	https://science.sciencemag.org/content/343/6175/1246980.long
Grundberg et al. (2016)	3	777	3.215	https://www.nature.com/articles/ng.2394
Ensembl Variant Effect Predictor	-	-	High impact	https://genomebiology.biomedcentral.com/articles/10.1186/s13059-016-0974-4
Ensembl Variant Effect Predictor	-	-	Moderate impact	https://genomebiology.biomedcentral.com/articles/10.1186/s13059-016-0974-4

Supplementary Table 4. Association results from genetic correlation analysis

Significant associations between the Bell's palsy meta-analysis and 600 published GWASs from the UK Biobank. *rG* genetic correlation. *SE* standard error.

Trait	rG	*P*	Z-Score (SE)
Number of treatments or medications taken	0.41	1.55×10^{-8}	5.66 (0.07)
Overall health	-0.36	1.79×10^{-7}	-5.22 (0.07)
Body mass index	0.26	1.52×10^{-6}	4.81 (0.05)
Age at first sexual intercourse	-0.28	2.80×10^{-6}	-4.68 (0.06)
Number of self-reported non-cancer illnesses	0.35	2.95×10^{-6}	4.67 (0.07)
Usual walking pace	-0.27	2.77×10^{-5}	-4.19 (0.07)
Endocrine diabetes	0.32	7.23×10^{-5}	3.97 (0.08)

Supplementary Table 5. Association results of rs9357446 with other diseases

Phenotype	Cohort	*P*	OR (95% CI)
Tuberculosis	Iceland, the UK, and Finland	0.012	0.97 (0.94, 0.99)
Systemic lupus erythematosus	Iceland, the UK, and Finland	0.11	0.94 (0.87, 1.01)
Multiple sclerosis	Iceland, the UK, and Finland	0.18	0.97 (0.92, 1.02)
Asthma	Iceland, the UK, and Finland	0.22	0.99 (0.98, 1.00)
Ulcerative colitis	Iceland, the UK, and Finland	0.41	1.01 (0.99, 1.03)
Type 1 diabetes	Iceland, the UK, and Finland	0.58	0.99 (0.94, 1.03)
Chronic obstructive pulmonary disease	Iceland, the UK, and Finland	0.77	1.00 (1.00, 1.00)
Rheumatoid arthritis	Iceland, the UK, and Finland	0.81	0.99 (0.95, 1.04)
Hypertension	Iceland and the UK	0.87	1.00 (1.00, 1.00)

Supplementary Note. rs9357446-A and RNA expression

rs9357446 is located on chromosome 6p21.1. We examined *cis*-eQTL in blood and adipose tissue in Iceland (Methods) and applied Bonferroni corrected *P* value threshold based on testing 65 genes in a 5 megabase window around the variant ($P < 0.05/65 = 7.69 \times 10^{-4}$). rs9357446-A significantly increases expression of Solute Carrier-Family 35 Member B2 (*SLC35B2*), located almost 222 kilobase upstream of the variant, in blood ($P = 2.20 \times 10^{-5}$, $\beta = 0.0540$) but not in adipose tissue ($P = 0.263$, $\beta = -0.0620$). However, another variant, rs28385699-C, is the top variant affecting the expression of *SLC35B2* ($P = 1.43 \times 10^{-71}$, $\beta = -0.517$), but does not significantly associate with Bell's palsy in the meta-analysis ($P = 1.72 \times 10^{-3}$, OR = 0.842). After adjusting the effect of rs9357446-A on *SLC35B2* for rs28385699-C, the *P* value is no longer significant ($P = 7.00 \times 10^{-3}$). Neither variant affected expression in any nearby genes in 18 other databases listed in Supplementary Table 3.

Supplementary Note. rs9357446-A and plasma proteomics

We tested associations between rs9357446-A and 4,983 plasma proteins measured in 35,559 Icelanders using SOMAscan (Methods). The strongest *trans*-pQTL association is with vascular endothelial growth factor (VEGF) (P = 1.06×10^{-6}, β = -0.042) encoded by *FLT1*. The two strongest *cis*-pQTL associations are with ectonucleotide pyrophosphatase/phosphodiesterase family member 5 (ENPP5) (P = 1.91×10^{-6}, β = 0.040) and cysteine rich secretory protein 2 (CRIS2) (P = 3.35×10^{-6}, β = -0.038) encoded by *ENPP5* and *CRISP2*, respectively. However, rs9357446 is not the top variant affecting these proteins. VEGF is involved in vasculogenesis and angiogenesis. ENPP5 may play a role in neuronal cell communication and CRIS2 may regulate activity of ion channels.
